# Maxillary Arch Intrusion With Temporary Anchorage Devices Versus Conventional Intrusion Arches: A Systematic Review and Meta-Analysis

**DOI:** 10.7759/cureus.109783

**Published:** 2026-05-27

**Authors:** Aparna Khamatkar, Aditi Sawtekar, Karthick Shetty, Veera Sawant, Sushma Sonawane, Sanpreet S Sachdev

**Affiliations:** 1 Department of Orthodontics and Dentofacial Orthopedics, D. Y. Patil Deemed to be University, School of Dentistry, Navi Mumbai, IND; 2 Department of Oral Pathology and Microbiology, Bharati Vidyapeeth (Deemed to be University) Dental College and Hospital, Navi Mumbai, IND

**Keywords:** deep bite, intrusion arch, maxillary incisor intrusion, orthodontic mini-implants, temporary anchorage devices

## Abstract

Deep bite correction often requires maxillary incisor intrusion, and both temporary anchorage devices (TADs) and conventional intrusion arches are used for this purpose. This systematic review and meta-analysis aimed to compare the effectiveness of TAD-supported maxillary arch intrusion with conventional intrusion arches in patients with deep bite or Class II malocclusion undergoing maxillary incisor intrusion.

A comprehensive electronic search was performed in PubMed, PMC/MEDLINE, Cochrane Library, and DOAJ for studies published up to December 31, 2025. Randomized controlled trials and non-randomized comparative clinical studies evaluating maxillary incisor intrusion with TADs versus conventional intrusion arches were included. Study selection, data extraction, risk of bias assessment, and quantitative synthesis were performed using predefined criteria. Nine studies met the eligibility criteria, including three randomized controlled trials and six non-randomized comparative studies. Both TAD-supported and conventional intrusion mechanics were effective in achieving maxillary incisor intrusion and reducing deep bite.

However, the pooled analysis favored TAD-supported intrusion for the rate of intrusion, amount of incisor intrusion, and overbite correction. The pooled standardized mean difference was 1.18 for the rate of intrusion, -0.88 for incisor intrusion, and -0.48 for overbite correction, all favoring TADs. Treatment duration did not differ significantly between the two approaches.

Qualitative findings also suggested better posterior anchorage preservation with TAD-supported mechanics, whereas conventional intrusion arches were more commonly associated with molar extrusion and incisor proclination. Within the limitations of the available evidence, TAD-supported intrusion tended to show favorable effects for some intrusion-related outcomes compared with conventional intrusion arches; however, treatment duration did not differ significantly, and adverse effects were also reported in some TAD protocols. The certainty of evidence was low to very low, and thus, further well-designed long-term studies are needed.

## Introduction and background

Deep bite is one of the most challenging vertical malocclusions in orthodontics because it may result from a combination of dentoalveolar overeruption, skeletal pattern, incisor inclination, curve of Spee depth, and soft-tissue influences [1]. Its correction, therefore, cannot be based on a single universal approach. In patients in whom growth modification is limited, or in those where dentoalveolar correction is the primary treatment strategy, treatment planning becomes more dependent on precise biomechanical control. In such situations, excessive maxillary incisor display may contribute not only to occlusal imbalance but also to functional and esthetic concerns such as gummy smile [2]. When a deep bite is primarily associated with maxillary incisor overeruption, true maxillary incisor intrusion becomes an important treatment objective.

Burstone’s classic biomechanical framework established that successful intrusion requires light continuous forces, careful control of the point of force application in relation to the center of resistance, and strict management of the posterior reactive unit to avoid undesirable effects such as molar extrusion or incisor flaring [3]. Conventional intrusion arches were developed from these principles and continue to be widely used because they permit controlled force delivery with relatively simple clinical application [4]. However, these mechanics primarily produce maxillary incisor intrusion and may be associated with reactive molar extrusion, depending on the force system and anchorage control [3,4]. These mechanics remain relevant in routine orthodontic practice, particularly when a noninvasive and economical approach is preferred.

The introduction of skeletal anchorage has significantly expanded the orthodontist’s ability to control vertical and sagittal side effects during intrusion mechanics. Temporary anchorage devices (TADs) can provide stationary anchorage, reduce dependence on patient compliance, and potentially minimize the posterior anchorage loss often associated with conventional mechanics [5]. At the same time, their clinical performance may be influenced by biologic stability, site of placement, and failure-related variables, and therefore their potential advantages should not be assumed without critical evaluation [6,7]. More broadly, previous evidence has shown that mini-implant anchorage can improve anchorage preservation compared with conventional methods, suggesting a possible benefit in intrusion therapy as well [8].

However, evidence specific to maxillary incisor intrusion remains less definitive. Previous systematic reviews have suggested that TAD-supported intrusion may improve upper incisor intrusion and reduce some adverse molar effects, but these conclusions were based on a limited number of studies and considerable methodological heterogeneity [9,10]. In addition, earlier reviews were broader in scope and did not focus specifically on maxillary incisor intrusion in direct comparison with conventional intrusion arches. An updated and focused comparison of TAD-supported maxillary incisor intrusion with conventional intrusion arches is therefore clinically relevant. The aim of the present systematic review and meta-analysis was to compare maxillary incisor intrusion achieved with TADs versus conventional intrusion arches. The specific objectives were to evaluate differences in intrusion amount, intrusion rate, overbite correction, treatment duration, and reported adverse outcomes.

## Review

Methodology

This systematic review and meta-analysis were undertaken to compare maxillary arch intrusion achieved with TADs and conventional intrusion arches in patients with deep bite or Class II malocclusion undergoing maxillary incisor intrusion. The review protocol was developed according to the Population, Intervention, Comparison, and Outcome (PICO) framework. The review was conducted in accordance with the PRISMA 2020 guidelines and was registered in the PROSPERO database (Registration ID: CRD42024553125) [11].

The focused review question was: “Is there a difference in maxillary arch intrusion achieved with TADs compared with conventional intrusion arches?” The population comprised patients with deep bite or Class II malocclusion undergoing maxillary incisor intrusion. The intervention of interest was intrusion using TADs, or orthodontic mini-implants. The comparison group included conventional intrusion arches such as the Connecticut intrusion arch, Burstone intrusion arch, and utility arch. The primary outcomes were the amount of intrusion, the rate of intrusion, overbite correction, and treatment duration. Secondary outcomes included reported adverse outcomes or complications associated with the intrusion mechanics.

Eligibility Criteria

Eligibility criteria were predefined according to the review question and PICO framework (Table 1). Studies were considered eligible if they included patients with deep bite or Class II malocclusion treated with maxillary arch intrusion using TADs and compared these with conventional intrusion arch mechanics. Randomized controlled trials and non-randomized controlled clinical studies published up to December 31, 2025, were eligible. Studies published in any language were considered if an English translation was available and the full text could be accessed. Studies were excluded if they were single-arm studies without a comparison group, review articles, case series, animal studies, abstract-only publications, or studies that did not report the required outcomes. Studies involving patients with previous orthodontic treatment or periodontal problems were also excluded.

**Table 1:** PICOS framework used to determine eligibility of the studies in the present systematic review

PICOS element	Inclusion criteria	Exclusion criteria
Population	Patients with deep bite or Class II malocclusion undergoing maxillary incisor intrusion	Patients not requiring maxillary incisor intrusion; patients with previous orthodontic treatment; patients with periodontal problems; animal studies
Intervention	Maxillary incisor intrusion using temporary anchorage devices (TADs) or orthodontic mini-implants	Studies not using TADs or mini-implants for maxillary intrusion
Comparison	Conventional intrusion arches, including the Connecticut intrusion arch, Burstone intrusion arch, utility arch, or other comparable intrusion mechanics	Studies without a comparison group; studies comparing interventions not relevant to conventional intrusion arch mechanics
Outcomes	Amount of intrusion, rate of intrusion, overbite correction, treatment duration, and reported adverse outcomes/complications	Studies not reporting the required outcomes or providing insufficient outcome data
Study design	Randomized controlled trials and non-randomized controlled clinical studies published up to December 31, 2025	Single-arm studies, case series, case reports, review articles, conference abstracts without full text, editorials, letters, and animal studies
Language/full-text availability	Studies published in any language, provided an English translation was available, and the full text could be accessed	Abstract-only publications; studies without accessible full text; studies without usable English translation

Information Sources and Search Strategy

A comprehensive electronic search was performed in PubMed, PubMed Central/MEDLINE, the Cochrane Central Register of Controlled Trials (CENTRAL), and DOAJ. The search strategy combined controlled vocabulary and free-text terms related to deep bite, Class II malocclusion, TADs, mini-implants, orthodontic implants, intrusion arches, Connecticut intrusion arch, Burstone arch, and utility arch. Boolean operators were used to combine the search terms appropriately. The final search covered all records available up to December 31, 2025, without language restrictions. The database-specific search yielded 21 records from PubMed, 195 from PMC/MEDLINE, 65 from Cochrane Central, and 136 from DOAJ. The main search concepts were based on population, intervention, comparator, and outcome terms, and the complete database-specific search strings are provided in Appendix 1. In addition, the reference lists of the eligible full-text articles were screened manually to identify any further relevant studies.

Study Selection

Two reviewers (A.S. and A.K.) independently screened the titles and abstracts of all retrieved records. Duplicate records were removed before screening. Potentially relevant studies were then selected for full-text evaluation, and eligibility was assessed against the predefined inclusion and exclusion criteria. Any disagreements between the two reviewers were resolved through discussion, and a third reviewer (K.S.) was consulted when necessary. Where clarification was required, attempts were planned to contact the study authors.

Data Extraction

Data extraction was carried out independently by two reviewers using a predesigned checklist. Discrepancies were resolved by discussion. The extracted variables included author and year, country, study design, sample size, age group, sex distribution, intervention details, comparator details, outcomes assessed, methods of outcome assessment, results, and study conclusions. Additional information on study setting, statistical methods, funding, and conflict of interest was collected where available. The extracted data were recorded in Excel sheets (Microsoft® Corp., Redmond, WA, USA) for outcome-wise analysis.

Risk of Bias Assessment

Methodological quality was assessed independently by two reviewers. Randomized controlled trials were evaluated using the Cochrane Risk of Bias 2 (RoB 2) tool [12]. Each domain and the overall study judgment were rated as low, some concerns, or high. Non-randomized comparative studies were assessed using the ROBINS-I tool, and the overall judgment was categorized as low, moderate, serious, or critical [13]. Any disagreements were resolved through discussion.

Data Synthesis and Statistical Analysis

A descriptive synthesis was first performed for all included studies. Quantitative synthesis was undertaken only when studies were considered clinically and methodologically comparable with respect to participants, interventions, comparators, and outcomes. The standardized mean difference (SMD) was used when the same construct was reported using different measurement approaches or scales across studies, whereas the mean difference (MD) was used when outcomes were reported using the same unit and scale. Patient-reported and complication-related outcomes were reported inconsistently across studies and were therefore synthesized narratively rather than quantitatively.

Clinical and methodological heterogeneity was considered before pooling the data. Statistical heterogeneity was assessed using the I² statistic, and values of 0%-30% were considered low, 30%-60% moderate, 50%-90% substantial, and 75%-100% considerable. Quantitative synthesis was considered appropriate only when heterogeneity was not judged to be excessive; studies with I² values greater than 75% were not considered suitable for pooled meta-analysis and were intended to be synthesized narratively. A random-effects model was used when heterogeneity was moderate to substantial, whereas a fixed-effects model was applied when heterogeneity was minimal. Statistical significance was set at p < 0.05.

Formal assessment of publication bias was not planned because fewer than 10 studies contributed to each pooled outcome. An exploratory subgroup-specific pooled analysis was undertaken for incisor intrusion by study design to examine whether the direction of effect remained consistent within randomized controlled trials. Owing to the limited number of studies in each category, these analyses were interpreted cautiously and used to support qualitative assessment of heterogeneity rather than definitive subgroup effect testing. Because of the limited number of studies within each pooled outcome and the heterogeneity in study design, comparator type, force magnitude, implant placement, and outcome assessment methods, other formal subgroup and sensitivity analyses were not feasible. However, a narrative subgroup-oriented synthesis was undertaken to explore patterns across major study and intervention characteristics.

Certainty of Evidence

The certainty of evidence for the outcomes included in the quantitative synthesis was assessed using the GRADE approach [14]. Each pooled outcome was evaluated across the domains of study limitations, inconsistency, indirectness, imprecision, and publication bias. Judgments were made separately for the rate of intrusion, the amount of incisor intrusion, overbite correction, and treatment duration, and the final certainty ratings were categorized as high, moderate, low, or very low.

Results

Study Selection

**Figure 1:**
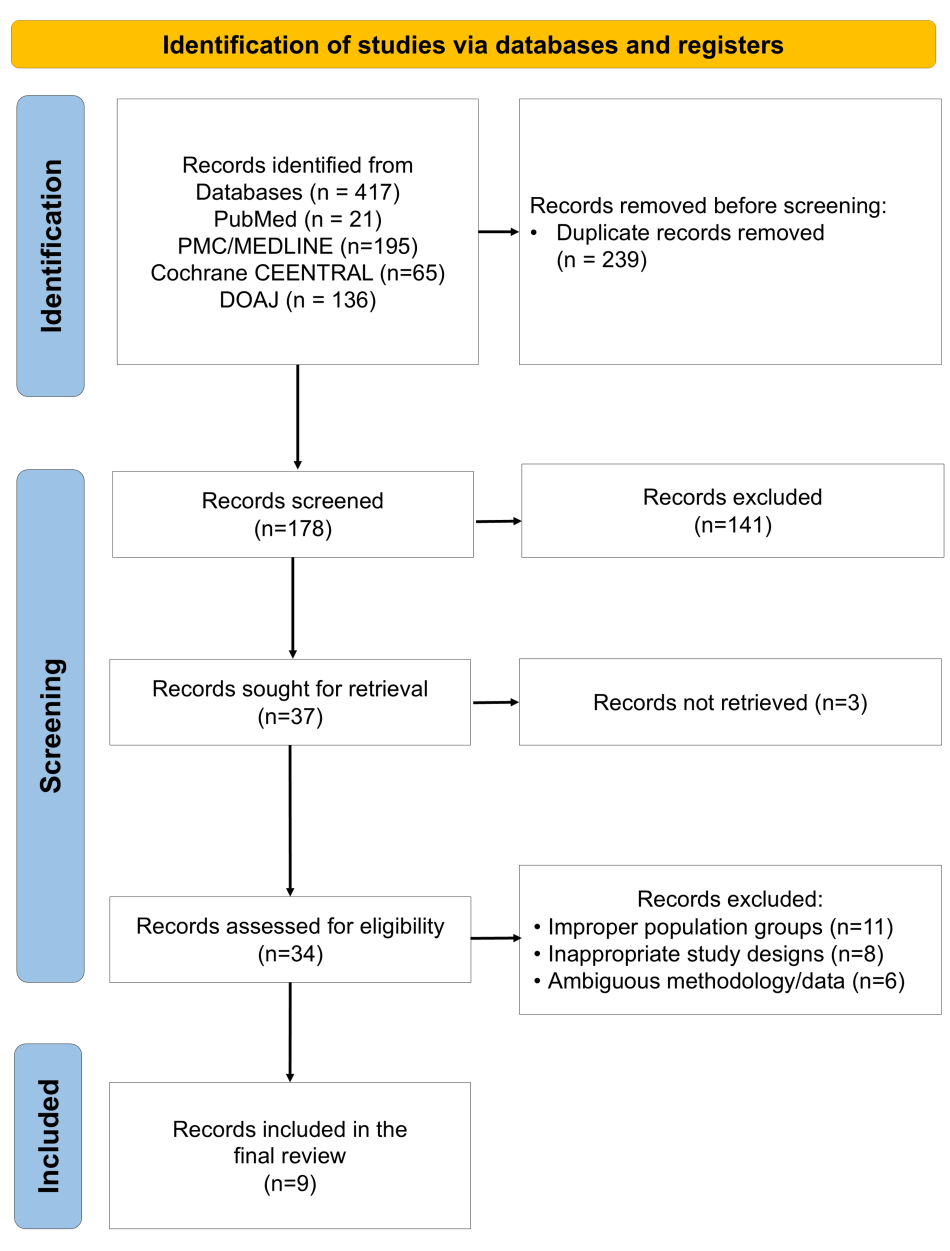
PRISMA flow diagram indicating the selection process of the records in the present systematic review

The electronic search identified 417 records across all databases. After removal of 239 duplicates, 178 records were screened by title and abstract, of which 141 were excluded as irrelevant. Thirty-seven reports were sought for retrieval, and three were not retrieved. The 34 retrieved full-text articles were assessed for eligibility. Twenty-five studies were excluded at the full-text stage because of different population characteristics (n = 11), inappropriate study designs (n = 8), or ambiguity in the data or methods (n = 6). Finally, nine studies met the eligibility criteria and were included in the qualitative synthesis and data analysis (Figure 1).

Characteristics of Included Studies

Nine comparative clinical studies were included [15-23]. Of these, three were randomized controlled trials and six were non-randomized controlled clinical studies [15-23]. The intervention arm in all included studies used temporary anchorage devices or mini-implants for maxillary incisor intrusion, whereas the comparison arm used conventional intrusion mechanics, most commonly the Connecticut intrusion arch; other comparators included Burstone intrusive arch, utility arch, and intrusive arch mechanics [15-23]. Lateral cephalometry was the principal method of outcome assessment in most studies, while one study used CBCT [15-23]. The commonly reported outcomes were the amount of intrusion, the rate of intrusion, overbite correction, treatment duration, and selected adverse effects such as root resorption, proclination, molar extrusion, pain, or tolerance [15-23]. The study characteristics are summarized in Table 2.

**Table 2:** Characteristics of included studies comparing TAD-supported and conventional maxillary incisor intrusion CBCT: cone-beam computed tomography; CTA: Connecticut intrusion arch; Div: division; F: female; M: male; MGJ: mucogingival junction; NR: not reported; RCT: randomized controlled trial; TAD: temporary anchorage device; TMA: titanium molybdenum alloy; y: years

Study	Country	Design	Sample	Age/sex	Key eligibility	TAD protocol	Comparator	Outcomes/tool	Main findings
Senışık and Türkkahraman (2012) [15]	Turkey	RCT	30 (15/15)	Age NR; 12 M, 18 F	Class II Div 2; overbite >4 mm; lower lip covering >4 mm of maxillary incisors	Self-drilling mini-implants, 1.3 × 5 mm; between lateral incisor and canine roots; 90 g/side	CTA; force NR	Incisor intrusion; cephalometric landmarks; lateral ceph	Both groups achieved maxillary incisor intrusion; TAD preserved anchorage better; CTA showed molar extrusion/anchorage loss
Jain et al. (2014) [16]	India	Prospective clinical	20 (10/10)	16-22 y; sex NR	Deep bite >4 mm; excessive incisal display at rest/smile	Two mini-implants, 1.4 × 6 mm; between central and lateral incisors; 1.5 oz	Ricketts utility arch; 1.5 oz	Incisor landmarks; lateral ceph	Both methods effective; TAD produced true intrusion with fewer side effects
Kumar et al. (2017) [17]	India	RCT	30 (15/15)	15-20 y; sex NR	Class II Div 1; overbite >6 mm	Microimplants, 1.3 × 7 mm; between central and lateral incisors; 60 g	CTA; 60 g	Rate of intrusion; lateral ceph	Intrusion greater with TAD; vertical molar change greater with CTA, but not significant
Raj et al. (2015) [18]	India	Prospective clinical	20 (10/10)	14-20 y; sex NR	Deep overbite ≥4 mm	Mini-implant; site NR; 70 g	Burstone intrusive arch; 70 g	Incisor intrusion; lateral ceph	Similar incisor intrusion; TAD gave true intrusion and better molar vertical control; intrusive arch showed molar extrusion
Ölmez Gürlen and Aras (2016) [19]	Turkey	RCT	32 (16/16)	Mean 14.5 y; 16 M, 16 F	Normal/increased vertical pattern; deep bite ≥5 mm	ORLUS mini-implants, 1.4 × 7 mm; between central and lateral incisors; 30 g	CTA; 60 g	Bite opening, root resorption, tipping; lateral ceph	Bite opening greater with TAD; root resorption and labial tipping greater with TAD
Kahraman et al. (2017) [20]	Turkey	Prospective clinical	34 (17/17)	Age NR; 9 M, 27 F	Supraerupted maxillary incisors; overbite >4 mm; gingival display ≥2 mm; incisor display at rest ≥3 mm	Miniscrews, 1.5 × 6 mm; between lateral incisor and canine; 80 g	CTA; 80 g	Molar effects; CBCT	Overall intrusive effects similar; TAD preferred when posterior anchorage preservation is needed
Gupta et al. (2017) [21]	India	Prospective clinical	24 (12/12)	15-25 y; sex NR	Deep bite ≥4 mm; maxillary incisor display >3 mm; no anterior trauma/loss	Two self-drilling mini-implants, 1.3 × 8 mm; between lateral incisor and canine; 30 g	CTA; force NR	Incisor intrusion; lateral ceph	Both methods effective; intrusion faster and greater with TAD; both caused unwanted tooth movements
El Namrawy et al. (2019) [22]	Egypt	Prospective clinical	30 (15/15)	17-29 y; 9 M, 21 F	Class I/II malocclusion; gingival display on smile; overbite ≥4 mm; maxillary incisor supraeruption	Two miniscrews, 1.4 × 6 mm; distal to lateral incisors at MGJ; 100 g	Intrusive arch, 0.017 × 0.025-in TMA; 100 g	Rate, skeletal/dental/soft-tissue effects, pain/tolerance; lateral ceph	Both methods effective; intrusive arch caused greater upper incisor proclination
Shakti et al. (2021) [23]	India	Prospective clinical	32 (10/10/12)	16-25 y; sex NR	Deep bite ≥4 mm; increased incisor/gingival display	Mini-implants, 1.3 × 8 mm; between lateral incisor and canine; 100 g	CTA (100 g) and Burstone 3-piece intrusion arch	Amount/rate of intrusion; lateral ceph	All 3 methods effective; no significant difference in amount or rate of intrusion

Qualitative Synthesis

Across the included studies, both TAD-supported and conventional intrusion mechanics were generally effective in intruding the maxillary incisors and reducing deep bite [15-23]. However, the overall pattern of treatment effects tended to favor TAD-supported intrusion for true incisor intrusion and posterior anchorage preservation [15-23]. Senışık and Türkkahraman reported that both methods achieved successful intrusion, but the Connecticut intrusion arch showed greater maxillary molar extrusion and loss of sagittal and vertical anchorage [15]. Kumar et al. reported a significantly greater amount and rate of intrusion with TADs [17]. Ölmez Gürlen and Aras found greater bite opening with mini-implants, although this was accompanied by increased labial tipping and root resorption [19]. Among the non-randomized studies, Jain et al. and Raj et al. reported that both modalities were effective, but TADs produced more genuine intrusion with better molar control [16,18]. Kahraman et al. found similar overall intrusive effects between methods, whereas Gupta et al. reported faster and greater intrusion with TADs [20,21]. El Namrawy et al. found both systems effective, but intrusive arch mechanics produced greater upper incisor proclination [22]. Shakti et al. reported no significant difference in the amount or rate of intrusion among mini-implants, Connecticut intrusion arch, and Burstone three-piece intrusion arch [23]. 

Narrative Subgroup Synthesis

By study design: When the findings were considered according to study design, both randomized and non-randomized comparative studies generally showed that TAD-supported mechanics were effective for maxillary incisor intrusion and tended to offer better anchorage control than conventional intrusion arches. Among the randomized trials, Senışık and Türkkahraman, Kumar et al., and Ölmez Gürlen and Aras all reported effective intrusion with TAD-supported mechanics, although some adverse effects such as labial tipping and root resorption were also noted in specific protocols [15,17,19]. Among the non-randomized studies, the overall pattern was similar, with several studies reporting more genuine intrusion, faster intrusion, or better molar control with TAD-supported mechanics, although the magnitude and consistency of these effects varied across studies [16,18,20-23].

By comparator type: When analyzed narratively by comparator type, most pooled and qualitative comparisons involved the Connecticut intrusion arch, which was the most frequently used conventional comparator [15,17,19-23]. Comparisons with Burstone intrusive arch or utility arch were fewer and more heterogeneous, which limited direct cross-study comparison [16,18,23]. Overall, the apparent advantage of TAD-supported mechanics was more consistently observed in studies using the Connecticut intrusion arch as the comparator.

By outcome assessment method: Most studies assessed outcomes using lateral cephalometry, whereas one study used CBCT to evaluate molar-related effects in greater detail [20]. Because CBCT-based assessment was reported in only one study, meaningful comparison by assessment method was limited. Nevertheless, the overall direction of findings remained broadly similar across assessment methods.

By force magnitude and implant placement: Force magnitude, mini-implant dimensions, and implant placement sites varied across the included studies [15-23]. These variations may have contributed to differences in the amount of intrusion achieved, anchorage effects, and adverse outcomes such as proclination or root resorption. However, the protocols were too heterogeneous and the number of studies too small to support formal subgroup analysis based on these variables. These subgroup observations were exploratory and descriptive only, and should not be interpreted as evidence of effect modification.

Risk of Bias

**Figure 2:**
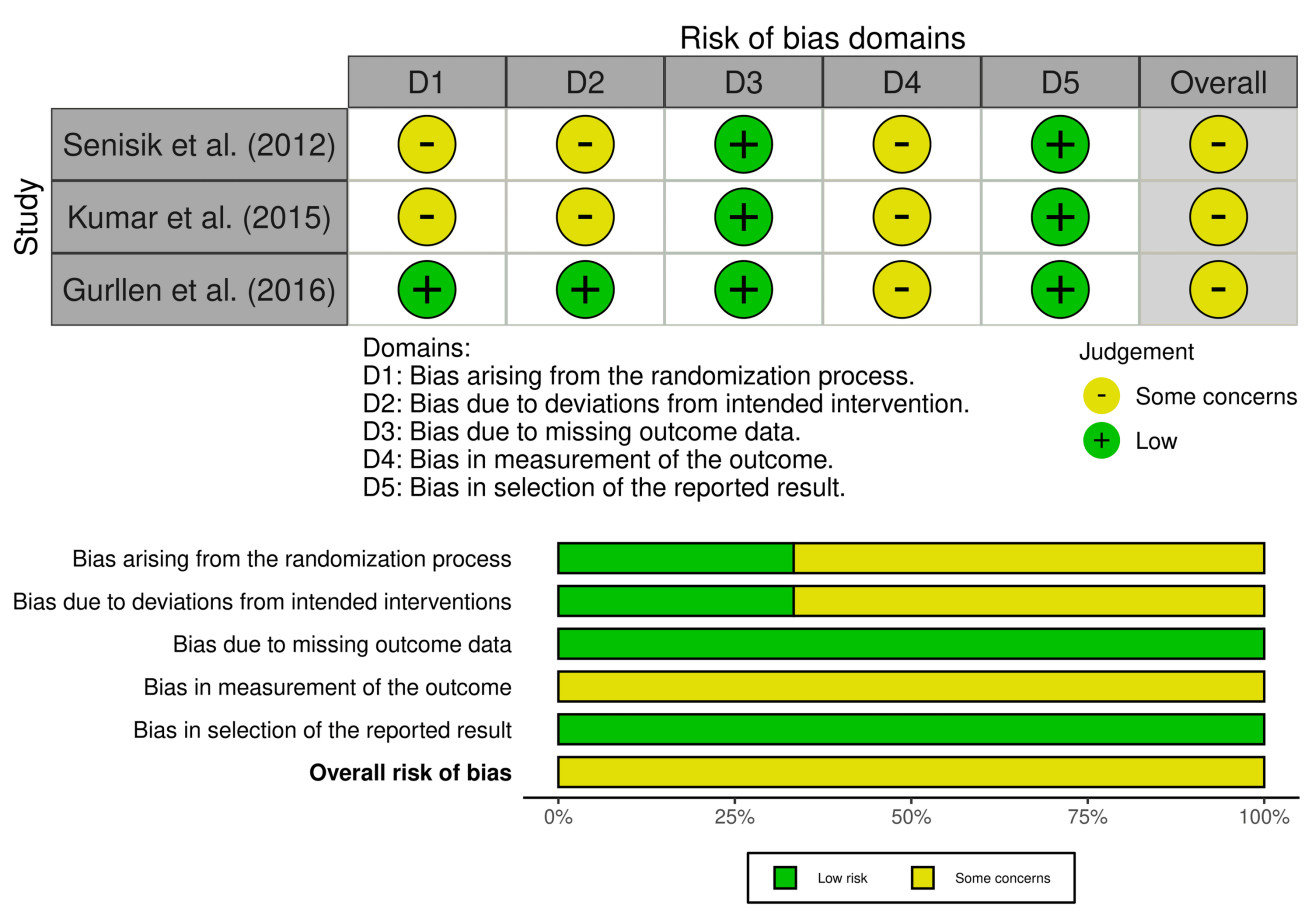
Risk of bias across the included randomized controlled trials using the Cochrane RoB-2 tool Source: [15,17,19]

Among the three randomized trials (Figure 2), all studies showed low judgment for missing outcome data and selection of the reported result [15,17,19]. Gürlen and Aras (2016) had low judgment for the randomization process and deviations from intended interventions, but some concerns regarding outcome measurement, leading to an overall judgment of some concerns [19]. Kumar et al. (2017) and Senışık and Türkkahraman (2012) showed some concerns mainly in the randomization process, deviations from intended interventions, and outcome measurement and were therefore also judged as having some concerns overall [15,17].

**Figure 3:**
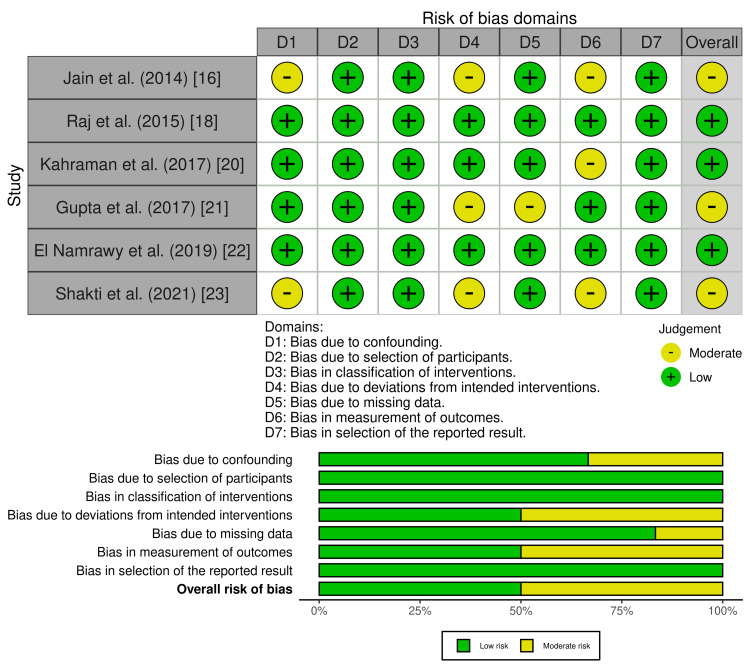
Risk of bias across the non-randomized clinical studies using ROBINS-I tool Source: [16,18,20-23]

Among the six non-randomized studies (Figure 3), Raj et al. (2015), Kahraman et al. (2017), and El Namrawy et al. (2019) were graded low overall, whereas Jain et al. (2014), Gupta et al. (2017), and Shakti et al. (2021) were graded moderate because of concerns related mainly to confounding, deviations from intended interventions, missing data, and outcome measurement [16,18,20-23]. Overall, the included evidence showed methodological limitations. The randomized trials were judged to have some concerns, and several non-randomized studies were rated as moderate, indicating that the findings should be interpreted cautiously [15-23].

Meta-Analysis

**Figure 4:**
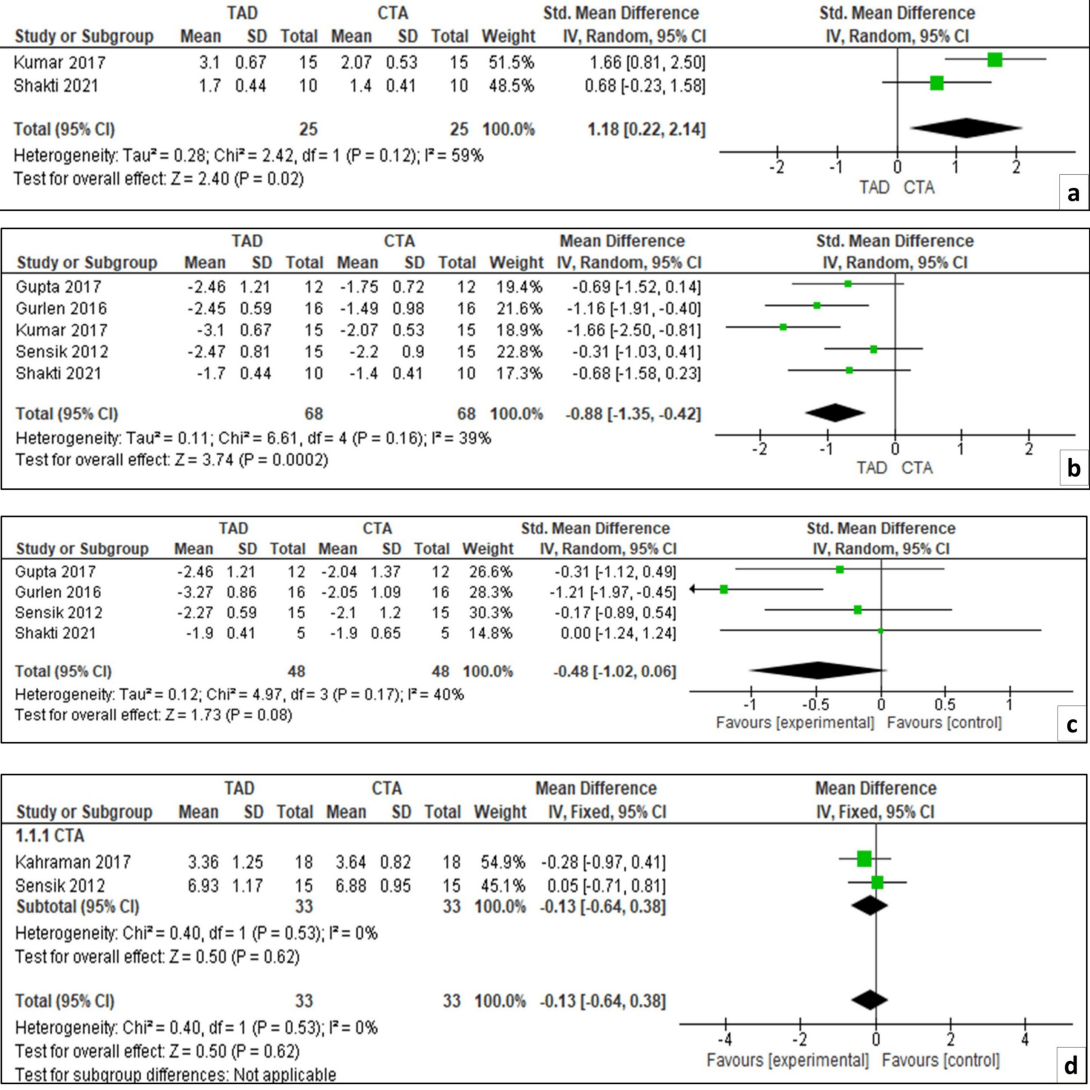
Forest plots showing comparison of TAD implants versus Connecticut intrusion arches in terms of (a) rate of intrusion, (b) incisor intrusion, (c) overbite correction, and (d) treatment duration Source: [15,17,19-23] TAD: Temporary anchorage device; CTA: Connecticut intrusion arches

Quantitative synthesis was performed for four outcomes using TADs as the intervention group and Connecticut intrusion arch as the comparator (Figure 4). For the rate of intrusion, two studies with 25 participants in each arm were pooled. The combined effect favored TADs, with an SMD of 1.18 (95% CI, 0.22 to 2.14), and the result was statistically significant. Heterogeneity was moderate (I² = 59%); therefore, a random-effects model was used [17,23].

For incisor intrusion, five studies with 68 participants in each group were included. The pooled SMD was -0.88 (95% CI, -1.35 to -0.42), indicating significantly greater intrusion with TADs than with the Connecticut intrusion arch. Moderate heterogeneity was present (I² = 39%), and a random-effects model was applied [15,17,19,21,23]. For overbite correction, four studies including 48 participants per group were analyzed. The pooled SMD was -0.48 (95% CI, -1.02 to 0.06), again favoring TADs. Heterogeneity was moderate (I² = 40%), and the random-effects model was retained [15,19,21,23]. For treatment duration, two studies contributed data. The pooled MD was -0.13 (95% CI, -0.64 to 0.38), suggesting a slightly shorter treatment duration with TAD-supported intrusion; however, this difference was not statistically significant. Because heterogeneity was minimal, a fixed-effects model was used [15,20].

**Figure 5:**
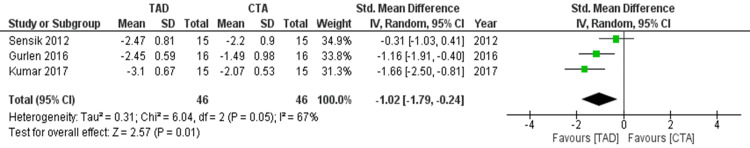
Subgroup pooled analysis of randomized controlled trials Source: [15,17,19] TAD: Temporary anchorage device; CTA: Connecticut intrusion arches

An exploratory subgroup-specific pooled analysis of randomized controlled trials was also performed for incisor intrusion (Figure 5). Three randomized studies were included in this subgroup analysis [15,17,19]. The pooled estimate favored TAD-supported intrusion, with an SMD of -1.02 (95% CI, -1.79 to -0.24), indicating greater incisor intrusion with TADs than with the Connecticut intrusion arch. Heterogeneity within this subgroup was substantial (I² = 67%), and the overall effect was statistically significant (p = 0.01). These findings suggest that the direction of effect in favor of TAD-supported intrusion was retained within the randomized evidence base, although the subgroup result should be interpreted cautiously because of the small number of studies and residual heterogeneity.

Overall, the quantitative synthesis showed that TAD-supported maxillary intrusion was associated with a significantly higher rate of intrusion, greater incisor intrusion, and better overbite correction than the CTA, while treatment duration did not differ significantly between the two approaches.

Certainty of Evidence

The GRADE assessment showed that the certainty of evidence ranged from very low to low across the pooled outcomes (Table 3). Evidence for the rate of intrusion and treatment duration was judged to be very low, primarily because of small sample sizes, limited numbers of contributing studies, and methodological concerns. Evidence for incisor intrusion and overbite correction was rated low, as the pooled effects were more consistent in favor of TAD-supported intrusion, but confidence in the estimates remained limited by study limitations and imprecision. Overall, although the meta-analysis favored TADs for key intrusion-related outcomes, the certainty of the current evidence remains limited.

**Table 3:** Certainty of evidence appraisal using GRADE approach * Publication bias could not be assessed formally because each pooled outcome included fewer than 10 studies.

Outcome	Studies	Participants	Pooled effect	Study limitations	Inconsistency	Indirectness	Imprecision	Publication bias	Certainty of evidence
Rate of intrusion	2	50	SMD 1.18 (95% CI 0.22 to 2.14)	Serious	Serious	Not serious	Serious	Undetected*	Very low
Incisor intrusion	5	136	SMD -0.88 (95% CI -1.35 to -0.42)	Serious	Not serious	Not serious	Serious	Undetected*	Low
Overbite correction	4	96	SMD -0.48 (95% CI -1.02 to -0.06)	Serious	Not serious	Not serious	Serious	Undetected*	Low
Treatment duration	2	NR	MD -0.13 (95% CI -0.64 to 0.38)	Serious	Not serious	Not serious	Serious	Undetected*	Very low

Discussion

Principal Findings

The present review suggests that both TAD-supported mechanics and conventional intrusion arches can effectively correct deep bite; however, the pooled estimates favored TADs for the rate of intrusion, the amount of true incisor intrusion, and overbite reduction [3,7,24]. This overall pattern is biomechanically plausible [3,24]. Conventional intrusion arches are effective when properly designed, but they depend on posterior dental anchorage and are therefore more vulnerable to reactive effects such as molar extrusion, molar tipping, and labial flaring of incisors if force application is not ideally controlled [3,24]. In contrast, TADs permit a more direct intrusive force system and reduce reliance on posterior teeth for anchorage, which helps explain the greater efficiency and anchorage preservation observed in many included studies [5,7,24].

Comparison With Previous Literature

These findings are also broadly consistent with prior secondary literature [4,9,10,25]. Earlier reviews reported that mini-implant-supported intrusion tends to produce greater true incisor intrusion and less molar extrusion than conventional mechanics, although the certainty of the evidence has generally remained low to moderate because of small trial numbers and methodological limitations [4,9,10,25]. The current review adds to that body of evidence by focusing specifically on maxillary arch intrusion relative to conventional intrusion arches and by showing that the advantage of TADs appears most consistent for biomechanical control rather than for shortening overall treatment time [4,25]. From a clinical perspective, this is important because, in deep-bite patients with excessive incisor display, stable vertical control and avoidance of posterior side effects may be more important than speed alone [1,24]. However, these findings should be interpreted in the context of the low- to very low-certainty evidence identified in the present review. This caution is particularly important because the evidence base included a small number of randomized trials together with non-randomized comparative studies, several of which had methodological limitations.

Clinical Implications

At the same time, the results do not imply that conventional intrusion arches are obsolete [3,5]. Burstone-type segmented mechanics and Connecticut intrusion arches remain useful, especially when the clinician seeks a noninvasive and lower-cost option or when mini-implant placement is limited by anatomy, patient preference, or operator experience [3,5,24]. Current biomechanical reviews emphasize that the choice of intrusion strategy should still be individualized according to smile line, incisor exposure at rest, vertical facial proportions, and the need for posterior anchorage control [1,5,24]. The exploratory pooled analysis restricted to randomized trials also favored TAD-supported intrusion, although heterogeneity remained substantial, indicating that differences in protocols and outcome assessment still influenced the subgroup estimate. Thus, the present findings suggest that TAD-supported mechanics may offer advantages for selected intrusion-related outcomes in some deep-bite cases, but these findings should be interpreted cautiously and should not be viewed as evidence of uniform superiority.

Adverse Effects and Biologic Considerations

An important point in this review is the issue of adverse effects, particularly root resorption and changes in incisor inclination. The included evidence did not demonstrate a uniform safety advantage for either approach. In fact, some studies reported greater labial tipping or greater root resorption with mini-implant-supported intrusion [19,26-28]. However, the proclination observed with intrusion arch mechanics should be interpreted as a treatment effect within specific reported protocols rather than as an unavoidable feature of all conventional intrusion mechanics. This is biologically credible because orthodontically induced inflammatory root resorption is influenced not only by appliance type but also by force magnitude, force duration, treatment mechanics, root morphology, and individual susceptibility [29,30]. Earlier work has shown that intrusive and retractive force systems can increase the risk of root resorption, and comparative studies of mini-implant-supported intrusion have similarly highlighted the need for careful radiographic monitoring during treatment [26-28].

Limitations and Future Directions

The interpretation of this review should therefore remain cautious. The included evidence base was relatively small, combined randomized and non-randomized designs, and showed variation in force magnitude, implant dimensions, comparator mechanics, cephalometric definitions, and follow-up duration. These sources of clinical and methodological heterogeneity may have influenced the pooled estimates and limited the strength of direct comparisons across studies. Blinding and allocation concealment were also incompletely reported in several studies. In addition, most studies provided only short-term treatment outcomes, whereas long-term stability after intrusion remains less well documented, particularly for TAD-supported mechanics [25]. Conventional utility-arch intrusion has shown encouraging long-term stability, but equivalent long-term evidence for TAD-based maxillary intrusion remains limited [31]. Future trials should therefore use larger sample sizes, standardized outcome definitions, and longer retention periods to assess both efficacy and post-treatment stability more confidently.

## Conclusions

Within the limitations of the available evidence, both TAD-supported mechanics and conventional intrusion arches were effective for maxillary incisor intrusion and deep-bite correction. Although the pooled findings tended to favor TAD-supported intrusion for the rate of intrusion and amount of incisor intrusion, treatment duration did not differ significantly between the two approaches, and adverse effects such as root resorption and tipping were also reported in some TAD protocols. Therefore, the current evidence suggests a possible advantage of TAD-supported intrusion for selected outcomes, but these findings should be interpreted cautiously because the certainty of evidence was low to very low, and further well-designed long-term studies are required before any definitive superiority can be established.

## Appendices

Appendix 1

**Table 4:** Search strings across individual databases

Database	Search	Number of articles obtained
PubMed	((maxillary arch intrusion OR maxillary molar intrusion) AND (temporary anchorage devices OR mini implants OR orthodontic mini implants)) AND (intrusion arches OR conventional intrusion arches OR Connecticut intrusion arch OR utility arch)	21
PMC/MEDLINE	((((((maxillary arch intrusion OR maxillary molar intrusion))) AND ((temporary anchorage devices OR mini implants OR orthodontic mini implants))) AND ((intrusion arches OR conventional intrusion arches OR Connecticut intrusion arch OR utility arch)))) AND ((prospective studies) OR retrospective studies)	195
Cochrane Central Library	Maxillary arch intrusion AND temporary anchorage devices OR mini implants AND intrusion arches OR conventional intrusion arches	65
DOAJ	Deep bite OR class II malocclusion OR deep overbite AND Temporary anchorage devices OR TADs OR mini implant OR orthodontic implants AND Intrusion arches OR Connecticut intrusion arch OR Burstone arch OR utility arch	136
